# Mutant CFTR Drives TWIST1 mediated epithelial–mesenchymal transition

**DOI:** 10.1038/s41419-020-03119-z

**Published:** 2020-10-26

**Authors:** Margarida C. Quaresma, Ines Pankonien, Luka A. Clarke, Luís S. Sousa, Iris A. L. Silva, Violeta Railean, Tereza Doušová, Jonas Fuxe, Margarida D. Amaral

**Affiliations:** 1 University of Lisboa, Faculty of Sciences, BioISI - Biosystems & Integrative Sciences Institute, C8 bdg, 1749-016 Campo Grande, Lisboa, Portugal; 2 Department of Paediatrics, 2nd Faculty of Medicine, Charles University and University Hospital Motol, V Uvalu 84, 15006 Prague, Czech Republic; 3 Division of Pathology, Department of Laboratory Medicine (LABMED), Karolinska Institutet and Karolinska University hospital, Huddinge, Stockholm Sweden

**Keywords:** Oncogenes, Respiratory tract diseases

## Abstract

Cystic fibrosis (CF) is a monogenetic disease resulting from mutations in the Cystic Fibrosis Transmembrane conductance Regulator (CFTR) gene encoding an anion channel. Recent evidence indicates that CFTR plays a role in other cellular processes, namely in development, cellular differentiation and wound healing. Accordingly, CFTR has been proposed to function as a tumour suppressor in a wide range of cancers. Along these lines, CF was recently suggested to be associated with epithelial–mesenchymal transition (EMT), a latent developmental process, which can be re-activated in fibrosis and cancer. However, it is unknown whether EMT is indeed active in CF and if EMT is triggered by dysfunctional CFTR itself or a consequence of secondary complications of CF. In this study, we investigated the occurrence of EMT in airways native tissue, primary cells and cell lines expressing mutant CFTR through the expression of epithelial and mesenchymal markers as well as EMT-associated transcription factors. Transepithelial electrical resistance, proliferation and regeneration rates, and cell resistance to TGF-β1induced EMT were also measured. CF tissues/cells expressing mutant CFTR displayed several signs of active EMT, namely: destructured epithelial proteins, defective cell junctions, increased levels of mesenchymal markers and EMT-associated transcription factors, hyper-proliferation and impaired wound healing. Importantly, we found evidence that the mutant CFTR triggered EMT was mediated by EMT-associated transcription factor TWIST1. Further, our data show that CF cells are over-sensitive to EMT but the CF EMT phenotype can be reversed by CFTR modulator drugs. Altogether, these results identify for the first time that EMT is intrinsically triggered by the absence of functional CFTR through a TWIST1 dependent mechanism and indicate that CFTR plays a direct role in EMT protection. This mechanistic link is a plausible explanation for the high incidence of fibrosis and cancer in CF, as well as for the role of CFTR as tumour suppressor protein.

## Introduction

Cystic fibrosis (CF) is the most common life-shortening monogenic condition in Caucasians, affecting over 90,000 individuals worldwide^1^. CF is a multi-organ disease, with manifestations that include pancreatic insufficiency, intestinal obstruction, liver disease and male infertility. However, its main cause of morbidity and mortality is respiratory failure due to pulmonary insufficiency, caused by chronic inflammation, recurrent infections and tissue fibrosis^2,3^. CF is caused by mutations in the gene encoding the CF Transmembrane conductance Regulator (CFTR) protein, a chloride (Cl^−^)/ bicarbonate (HCO_3_^−^) channel expressed at the apical plasma membrane (PM) of epithelial cells^4^. The most common CF-causing mutation is deletion of phenylalanine 508 (F508del), which leads to defective intracellular trafficking to the PM^5^. Other CFTR mutations cause decreased protein production, defective channel gating or conductance, decreased stability at the PM or total absence of CFTR protein^1^.

Besides its major function as an anion channel and regulator of other epithelial channels^2^, CFTR has also been implicated in other cellular processes including epithelial differentiation, polarization and proliferation. Indeed, CFTR has been related to the normal organization and function of tight junctions (TJs) and its presence at the PM was described as essential for epithelial tightness^6^. Functional CFTR is also required for rapid regeneration of human airway surface epithelium after injury, and wound healing is delayed in CF epithelia compared with non-CF controls^7,8^. CF vs non-CF airway epithelia also exhibit an overall delay in differentiation^3^ and dysfunctional CFTR leads to morphological abnormalities in proximal airways during early foetal airway development^9^. Indeed, mice, pigs and young children lacking functional CFTR display defects in tracheal development namely, malformations of the cartilaginous rings, reduced breathing rate and decreased contractile response^10,11^. Another CF airways feature is increased epithelial cell proliferation^3,12^. Significantly, a longitudinal study of a large cohort of individuals with CF reported higher risk of several cancer forms, especially of the digestive tract^13^. Many studies have reported that CFTR functions as a tumour suppressor^14–16^, and some suggested a link between CFTR downregulation in tumour cells and induction of epithelial–mesenchymal transition (EMT)^17–19^. Recent transcriptional profiling analysis revealed impaired epithelial differentiation and an EMT signature in CF airways^20^. However, it is unknown whether EMT is indeed active in CF and if so, what is the trigger, dysfunctional CFTR or a secondary disease event (e.g., chronic inflammation). Importantly, the mechanisms by which CFTR regulates development, differentiation or even tumorigenesis are poorly understood. Recently, the possible relationship between CFTR and EMT was comprehensively reviewed^21^.

EMT is a latent, developmental process, involving transcriptional reprogramming of epithelial cells into a mesenchymal phenotype with enhanced migratory properties. The EMT programme is driven by EMT-associated transcription factors (EMTa-TF) including members of the Snail, Zeb and Twist families. During EMT, genes encoding epithelial-specific proteins including components of tight (TJ), adherens (AJ), gap (GJ) junctions, and desmosomes are inactivated, and the cytoskeleton reorganized. Consequently, apical-basal polarity is lost, and cell shape changes. In parallel, genes defining the mesenchymal phenotype including those associated with the cytoskeleton (vimentin and αSMA), cell junctions (N-cadherin) and secreted extracellular matrix proteins (ECM, collagen I and fibronectin) are activated^22,23^. Developmental EMT (usually called type 1) is critical for tissue development and organogenesis but is silent in normal, healthy adult tissues. However, EMT reactivation occurs in certain pathological conditions including inflammatory diseases (type 2), or cancer (type 3)^24^. Cancer-related EMT contributes to tumour progression into invasive and metastatic disease and has also been linked to cancer stem cells and chemoresistance^25^.

EMT is also a driver of tissue remodelling and fibrosis in inflammatory diseases, which was recently shown to be active in several chronic lung diseases, including chronic obstructive pulmonary disease (COPD) and idiopathic pulmonary fibrosis (IPF)^26–28^. An important EMT trigger is TGF-β1, which is overexpressed in these chronic airway diseases, and also in CF^29^. CF shares some clinical features with COPD and IPF (obstructed airways, chronic inflammation and fibrosis) and gene expression profiles^20^. Of note, in these diseases, EMT is present as hybrid or partial EMT: cells display intermediate epithelial/mesenchymal states^26–28^. Along these lines, current evidence suggests that EMT should be regarded as a spectrum of dynamic yet stable states instead of a binary decision, with cells having substantial plasticity and possibly co-expressing epithelial and mesenchymal phenotypes. In fact, epithelial cells that have activated an EMT program very rarely advance to a fully mesenchymal state, expressing instead a mixture of markers^25,30^.

Our aim here was to investigate whether dysfunctional CFTR triggers EMT and if this process plays a role in CF lung disease progression. Our findings clearly show that EMT is present in native CF bronchial tissues and cells by several lines of evidence. Firstly, epithelial proteins are mislocalised/disorganized in CF tissues/cells and mesenchymal markers are significantly increased vs controls. Secondly, CF cells exhibit significantly reduced transepithelial electrical resistance (TEER) values, consistent with impaired barrier function and TJ defects. Thirdly, CF cells display increased proliferation and decreased wound healing. Fourthly, EMTa-TFs levels are consistently increased in CF tissues and cells, with TWIST1 expression showing a direct link to dysfunctional CFTR. Fifthly, CF cells are over-sensitive to TGF-β1-induced EMT. Finally, the epithelial phenotype in CF cells can be restored by drugs rescuing the traffic and function defects of the most frequent mutant, F508del-CFTR. Altogether, these data indicate for the first time that absence of functional CFTR leads to (partial) EMT, that this process is mediated by TWIST1 and that functional CFTR confers some degree of EMT protection (e.g. to cancer). This mechanistic link is a plausible explanation for the fibrosis phenotype and higher cancer incidence in CF, unravelling a novel carcinogenesis pathway requiring investigation and targeting.

## Materials and methods

### Native human lung tissue

Explanted CF lungs and control tissues were collected in the Paediatrics Department of Motol University Hospital (Prague, Czech Republic) under approval of applied regulations and the hospital’s Ethics Committee and shipped over 24 h to Lisboa. Informed consent was obtained from all subjects. After cleaning, primary, secondary and tertiary bronchi were used for primary HBE (pHBE) cell isolation, RNA extraction and immunohistochemistry. For RNA extraction, pieces of secondary bronchi were collected after lung cleaning and were included in extraction buffer (see qRT-PCR), snap frozen in liquid nitrogen and kept at −80 °C. For immunohistochemistry, tissue was fixed overnight with electron microscopy grade PFA (0.2% v/v, Electron Microscopy Sciences, 15710) and then dehydrated and slowly frozen as previously described^31^. Briefly, after lung cleaning and fixation, the pieces of secondary/tertiary bronchi were kept for 12 h at a time in phosphate buffers with increasing sucrose (Fluka, 84100) content (4–15%) and then incubated in a final solution with 15% sucrose and 7.5% gelatine (Sigma-Aldrich, G9391) for 1 h at 37 °C. Dry ice-chilled isopentane (VWR, 24872) was then used to slowly freeze the tissues, which were kept at −80 °C until sectioning. Tissue sections were cryocut using a Leica CM1850 UV cryostat. Cryosections 5–10 μm thick were generated on silane-prep slides (glass slides coated with aminoalkylsilane, Sigma-Aldrich, S4651), left to dry overnight at 37 °C and used the following day for immunohistochemistry.

### Primary human bronchial epithelial cells

Primary human bronchial epithelial (pHBE) cells were isolated as previously described^32^. Briefly, cells were dissociated from the bronchial tissue by protease/DNase treatment and were then collected by centrifugation at 500 g for 5 min at 4 °C. PHBE cells were cultured in BEpiCM (ScienceCell, 3211) in six-well culture plates previously coated with PureCol (type I collagen) (30 μg/mL, Advanced Biomatrix, 5005). To generate a differentiated epithelium 2 × 10^5^ cells were seeded onto collagen IV (Sigma-Aldrich, C7521) pre-coated Transwell 6.5 mm permeable supports (Corning, 3470) and, when confluent, culture medium was removed from the apical surface. Cells were kept on ALI for 21 days in order to fully differentiate before experiments (e.g. Western blot, wound healing) were performed. Growth curves were performed on non-polarized HBE cells plated in PureCol coated 24-well culture plates.

CF cells with three different genotypes were used throughout this work: F508del/F508del, R347P/711 + 5 G > A and M1101K/1609delCA. These genotypes comprise different mutation classes, i.e. the mutations affect CFTR function differently. 1609delCA is a frameshift mutation generating a premature stop codon e.g., a class I mutation^33^. F508del and M1101K are class II mutations, affecting CFTR traffic, and leading to ER retention^34^. R347P, a class IV mutation, causes a decrease in CFTR conductance^35^. Finally, class V mutation 711 + 5 G > A leads to alternative splicing resulting in a lesser amount of normally spliced CFTR transcripts^36,37^.

### Cell lines

CF-relevant immortalized bronchial epithelial cell lines, CFBE41o- (Cystic Fibrosis Bronchial Epithelial) cells stably overexpressing wt- and F508del-CFTR^38^, were used in this work. CFBE cells were grown in Minimum Essential Medium Eagle (MEM) with Earl salts and L-glutamine (Corning, 10–010-CVR) supplemented with 10% (v/v) Foetal Bovine Serum (FBS) (Gibco, 10270), 1% Pen/Strep and puromycin (Sigma-Aldrich, P8833) at 2.5 μg/mL for selection. All cell lines tested negative for mycoplasma. To achieve polarization, cells were seeded on collagen IV pre-coated Transwell permeable supports at a density of 1.25, 2.5 or 10 × 10^5^ cells, depending on the diameter of the filter (6.5 mm, 12 mm or 24 mm insert, Corning 3470, 3460 and 3450, respectively). On the following day, media was changed from 10% to 2% (v/v) FBS to promote differentiation/polarization. The transepithelial electrical resistance (TEER) was measured at regular time intervals.

### TEER measurements

TEER measurements were carried out in polarizing CFBE cells using a volt-ohmmeter (Millicell-ERS, Millipore, MER5000001), as a first indicator that the cells were differentiated and ready for further experiments, and to confirm re-differentiation after wound healing. TEER measurements were also carried out in the Ussing chamber, namely in pHBE cells after 21 day in ALI culture.

### TGF-β1 and CFTR modulator treatments

Polarized CFBE cells were incubated 3 days after seeding with 15 ng/mL human TGF-β1 from HEK293 (human embryonic kidney) cells (PeproTech, 100–21) for 48 h. A negative control with the solvent (10 mM citric acid (Sigma-Aldrich, 251275) (pH 3) and 0.1% bovine serum albumin (BSA) (Sigma-Aldrich, A9647) was included in all experiments.

In addition, 3 μM VX-445 (SelleckChem, S8851), 5 μM VX-661 (SelleckChem, S7059) and/or 3 μM VX-770 (SelleckChem, S1144) were added to polarized CFBE cells on day 3 after seeding for 24 h. Compounds were dissolved in DMSO which was used as a negative control in the experiment.

### TWIST1 shRNA knockdown

HEK 293T cells were used to produce lentiviral particles containing shTWIST1 (Sigma-Aldrich, TRCN0000020543) and shLuciferase (Sigma-Aldrich, SHC007) (as a negative control). HEK cells (5 × 10^5^ cells per well) were transfected with 5 µg of DNA (per well)—2.38 µg of packaging plasmid pCMV-dR8.74psPAX2, 0.24 µg of envelope plasmid VSV-G/pMD2.G, and 2.38 µg of the shRNA plasmids. Cells were then incubated for 18 h after which medium was replaced to remove the transfection reagent and the cells were incubated for an extra 30 h. The media containing the lentiviral particles were harvested, mixed with PEG-it Virus Precipitation Solution (System Biosciences, LV810A-1) and left overnight at 4 °C. The harvested viral particles were then used to transduce CFBE wt- and F508del-CFTR cells. CFBE cells were infected with 1.5 mL of lentivirus-containing medium. The plates were centrifuged at 200×*g* for 1 h at 25 °C and then incubated for 24 h at 37 °C, 5% CO_2_. The medium was then changed to the respective cell medium supplemented with selection antibiotics to eliminate the non-transduced cells.

### Immunofluorescence staining (IF)

Polarized CFBE cells were fixed with PFA (Merck Millipore, 104003) 4% (v/v), permeabilized with triton X-100 (Amersham Biosciences, 17–1315–01) 0.5% (v/v) and blocked with BSA 1% (w/v) before being removed from their supports using a scalpel. Cells were then incubated overnight at 4 °C with primary antibodies, after which a mix of the secondary antibodies and nuclear dye (4 μg/mL, Methyl Green, Sigma-Aldrich, 67060) was applied for 2 h at RT. Filter sections were mounted in a mix of N-propylgallate (Sigma-Aldrich, P3130) and Glycerol for microscopy (Merck, 104095).

Lung tissue stainings were performed similarly but permeabilization was achieved with a 0.2% (v/v) triton X-100 solution and a quenching step with NaBH_4_ (1 mg/mL, Sigma-Aldrich, 213462) was additionally performed before blocking. Hoechst 33258 (1 μg/mL, Sigma-Aldrich, 94403) was used to stain the nuclei. The tissues stained were secondary/tertiary bronchi and were as similar as possible for comparison. Areas of extensive shedding/remodelling in CF tissue were avoided in the analysis, and areas of intact epithelia preferred. Maintenance of the correct architecture of the epithelia by the cryopreserving protocol was confirmed by detecting several cell-specific markers in control trachea (Fig. S1).

Imaging was performed with a Leica TCS SP8 confocal microscope coupled to a Hamamatsu Flash4 sCMOS camera, using HC Plan Apo 20×/0.75 and HC Plan Apo 63×/1.4 objectives. Software used for acquisition was Leica’s LAS x, and image processing was performed on ImageJ FIJI^39^. FIJI was used to generate maximum image projections, isolate individual z-slices and produce orthogonal views by re-slicing the z-stacks.

A list of primary and secondary antibodies can be found in Tables S1,S2.

### qRT-PCR

Bronchial samples from four CF individuals (F508del/F508del) and from four non-CF controls were used in transcript analysis. RNA was extracted using the NZY Total RNA Isolation kit (Nzytech, MB13402). After lung cleaning, pieces of secondary bronchi were collected and kept in NR buffer at −80 °C. Upon thawing the extraction was carried out as indicated by the manufacturer. cDNA was generated with NZY M-MuLV Reverse Transcriptase (Nzytech, MB08301). Quantitative reverse transcription polymerase chain reaction (qRT-PCR) was performed as previously described^40^. Briefly, a mix containing forward and reverse primers, cDNA (5 ng) and 1x Evagreen SsoFast PCR reagent (Bio-Rad, 172–5204) was used along with a Bio-Rad CFX96 system. Bio-Rad CFX Manager 2.0 software (Bio-Rad, 1845000) was used for analysis. Mean relative transcript levels were calculated by normalizing the gene of interest against the control endogenous gene (GAPDH) and applying the ΔΔCT method where ${\mathrm{Fold}}\;{\mathrm{Change}}\left( {{\mathrm{FC}}} \right) = 2^{ - \Delta \Delta {\mathrm{CT}}}$. The mean CT (cycle threshold) of the control gene is subtracted from the mean CT of the target gene, giving the ΔCT. The mean ΔCT is then calculated for CF samples gene expression relative to control lung ΔCT (ΔΔCT). A standard cycle protocol was used for PCR amplification (1 min at 95 °C followed by 40 cycles of 10 s at 95 °C and 30 s at 60 °C). Sequences for the primers were found at Harvard Primerbank (Table S3). Melt curves confirmed amplification of unique specific products.

### Western blot

Western blot (WB) analysis of cell lysates was performed as previously described^41^. Briefly, CFBE or pHBE cells grown on Transwell inserts were washed twice with cold PBS and lysed with a buffer containing 31.25 mM Tris HCl (Sigma, 30721) pH 6.8; 1.5% (v/v) sodium dodecyl sulphate (SDS) (Gibco, 15553); 10% (v/v) glycerol (Sigma, 92025); 50 mM dithiothreitol (DTT) (Sigma, D0632) and protease inhibitor cocktail (Roche, 11697498001). Benzonase (Sigma-Aldrich, E1014) 25 U/mL was also added to shear the DNA. In all, 25–30 μg of protein were loaded onto polyacrylamide gels (4% for stacking and 7%, 10% or 12.5% for resolving gels) in order to perform SDS/PAGE. Transfer onto polyvinylidene difluoride (PVDF) membranes (Merck Millipore, IPVH00010) was preformed using a wet-transfer system. The membranes were blocked for 1 h with 5% (w/v) non-fat milk (NFM) in PBS supplemented with Tween 20 (Fisher BioReagents, BP337–100), or, in particular cases (see Table S1), 1% (w/v) NFM in Tris buffer saline with Tween 20. This was followed by incubation with the primary antibody overnight at 4 °C, with gentle shaking. Horseradish peroxidase (HRP)-conjugated secondary antibodies were applied for 1 h at RT. All the antibodies were diluted in the blocking solution. Membrane luminescence was detected on a Chemidoc XRS + system (BioRad, 170–8265). Quantification of band intensity was performed using the Image Lab software (BioRad, 170–9690), which integrates peak area. All measurements were normalized against loading controls (calnexin, tubulin or GAPDH). A list of primary and secondary antibodies can be found in Tables S1 and S2.

### Growth curve

Control and CF pHBE cells were seeded in 24-well plates previously coated with PureCol at a density of 50,000 cells/well (day 0). Cells were kept in BEpiCM and were harvested and counted every 2–4 days to assess the growth rate. Media was changed regularly. At least three different wells were considered for all time points in all controls and CF pHBE cells.

### Wound healing

Fully polarized CFBE cells or fully differentiated pHBE cells were mechanically injured by scraping a sterile P10 pipette tip across the cell monolayer. For cell wounding PBS was added to the apical side of the filters. After wounding the apical surface was washed twice with PBS to remove cell debris. Fresh media was added to the basolateral (on both cell types) and apical side (only on CFBE cells).

Wound closure was monitored by live cell imaging (48 h, 37 °C, 5% CO_2_) with an automated Leica DMI6000 widefield microscope coupled to a Hamamatsu Flash4 sCMOS camera, using a HCX 4x W 4×/0.1 objective. Images were taken every 2–3 h. Software used for acquisition was Leica’s LAS x, and image processing was performed on ImageJ FIJI^39^. FIJI was used to segment, and measure wound area. Wound closure was then calculated as ${\mathrm{Wound}}\;{\mathrm{size}}\left( \% \right) = \left( {A_t/A_0} \right) \times 100$, where *A*_*t*_ is the area for a given time point and *A*_0_ is the initial wound area. Wound size was plotted as a function of time (h) and was used to calculate the rate (slope) of wound closure (%/h).

### Antibodies and primers

A list of primary and secondary antibodies used in both IF and WB can be found in Tables S1 and S2, respectively. Sequences for the primers used in qRT-PCR are listed in Table S3.

### Statistical analyses

Data are presented as mean ± S.E.M, with number of replicates described in the legend of each figure. Data points were considered outliers and excluded if their value was four times greater or smaller than the other replicates. Two-sided student’s t-test for unpaired samples was used for statistical analyses (comparison of the differences in the means between two groups with small number of replicates (*n* < 30)). Normal distribution of the data was assumed, and the variance was similar between groups. Both the selected sample sizes and the statistical analysis chosen are standard procedure for the experiments conducted in this work. Prism 6 software (GraphPad, Inc., San Diego, CA) was used for graph design and statistical analyses. Significant differences were defined for *p* ≤ 0.05.

## Results

### Native human CF airways display EMT characteristics

Native CF and control bronchial tissue was investigated by a combination of transcript and immunofluorescence (IF) analyses using established EMT markers to provide insight into this process in CF.

**Fig. 1:**
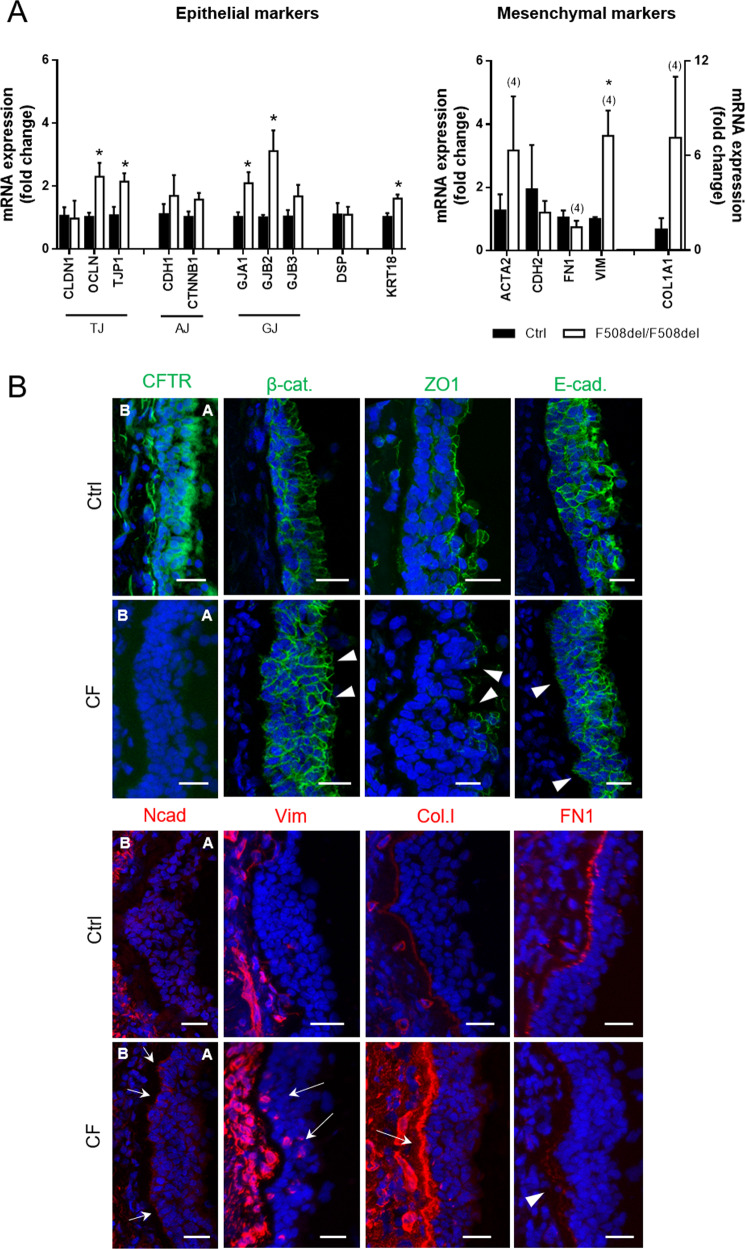
CF bronchial tissue presents disorganized/mislocalized epithelial markers and upregulated mesenchymal markers when compared to control. **A** F508del/F508del individuals show upregulation of several epithelial and mesenchymal genes. QRT-PCR was performed on different control and F508del homozygous lung samples. Fold-change in mRNA expression levels was assessed for different epithelial and mesenchymal markers. These comprised markers of several cell junctions as well as the cytoskeleton and the ECM. Fold-change values were calculated by applying the ΔΔCT method and are represented by mean ± SEM relative to the control samples (*n* = 3, unless indicated by (4)). Asterisk indicates significant differences between F508del/F508del and Ctrl samples (unpaired *t*-test, *p* < 0.05). **B** Representative images of native human bronchial tissue (Ctrl and CF) immunostained for epithelial (CFTR, β-catenin, ZO1, E-cadherin) and mesenchymal (N-cadherin, vimentin, collagen I, fibronectin) markers. Nuclei are depicted in blue and proteins in green (epithelial) or red (mesenchymal). Scale bar represents 25 μm. Disrupted or abnormal epithelial markers and FN1 (arrowheads) and increased mesenchymal markers (arrows) are observable in CF tissue when compared to controls. Apical and basal (A and B, respectively) sides of the epithelia are identified and are the same in all pictures. A Leica TCS SP8 confocal microscope was used for image acquisition. CFTR, ZO1, Vim, Col.I and FN1 are displayed as maximum image projections (MIPs) and β-cat, E-cad and N-cad as individual z-stacks. The tissues stained display secondary/tertiary bronchi and were as similar as possible for comparison. Areas of extensive shedding/remodelling in CF tissue were avoided, and areas of intact epithelia preferred. The CF individual in this figure had a R347P/711 + 5 G > A genotype, but similar findings were obtained for the F508del/F508del genotype, demonstrating that the phenotype was not restricted to any genotype. Several controls were also assessed with similar results between themselves. (*n* = 2–3 samples).

Transcript analysis showed no differences in mRNA levels between F508del-homozygous and control lung specimens for the epithelial markers claudin-1 (CLDN1), E-cadherin (CDH1), β-catenin (CTNNB1), connexin 31 (GJB3) and desmoplakin (DSP) (Fig. 1A). However, the mRNA levels of occludin (OCLN), tight junction protein 1/zonula occludens-1 (TJP1/ZO-1), connexin 43 (GJA1), connexin 26 (GJB2), and cytokeratin 18 (KRT18) were significantly increased in CF vs control tissue. No difference was observed in the transcripts of mesenchymal markers N-cadherin (CDH2) or fibronectin (FN1) (Fig. 1A). In contrast, vimentin (VIM) was significantly increased in CF vs control tissue. A tendency towards increased levels in CF tissue was also detected for α-smooth muscle actin (ACTA2/α-SMA) and collagen I α−1 (COL1A1).

IF staining and confocal microscopy were used to evaluate protein expression and localisation of EMT markers in CF versus control tissue and revealed that, as expected, CFTR was not detected in CF tissue, in contrast to CFTR apical localisation in control tissue (Fig. 1B). The AJ component β-catenin (β-cat) localized to cell junctions in both CF and control tissue, but evidenced structural epithelial architecture differences between these tissues revealing the presence of polygonal flat cells on the CF epithelial layer surface and also fewer cylindrical-shaped columnar cells and several cell layers in CF tissue (Fig. 1B). This is indicative of a transitory state between pseudostratified structure and squamous metaplasia^42^, indicating that the pseudostratified epithelium, characteristic of the bronchi, is compromised in CF. ZO-1 was detected at TJs in both CF and control tissue, but the staining showed various gaps in the CF epithelium (Fig. 1B). E-cadherin (E-cad) was present both in CF and control tissues, but appeared weaker in the basal cell layers of the CF epithelium vs control (Fig. 1B), which stained positively for N-cadherin, vimentin and collagen I, being absent in control (Fig. 1B). In contrast, a well-defined layer for fibronectin (FN1) was observed in control but not in CF tissue (Fig. 1B).

This CF tissue has from an individual with the R347P/711 + 5 G > A genotype, but similar findings were obtained for F508del/F508del tissue (data not shown), demonstrating that this phenotype is not restricted to a given genotype. Altogether, these results indicate disruption of epithelial and induction of mesenchymal markers in CF vs control tissue, thus indicative of EMT. However, overall downregulation of epithelial markers was not detected, suggesting partial EMT.

### Primary CF cells are more mesenchymal than non-CF cells

**Fig. 2:**
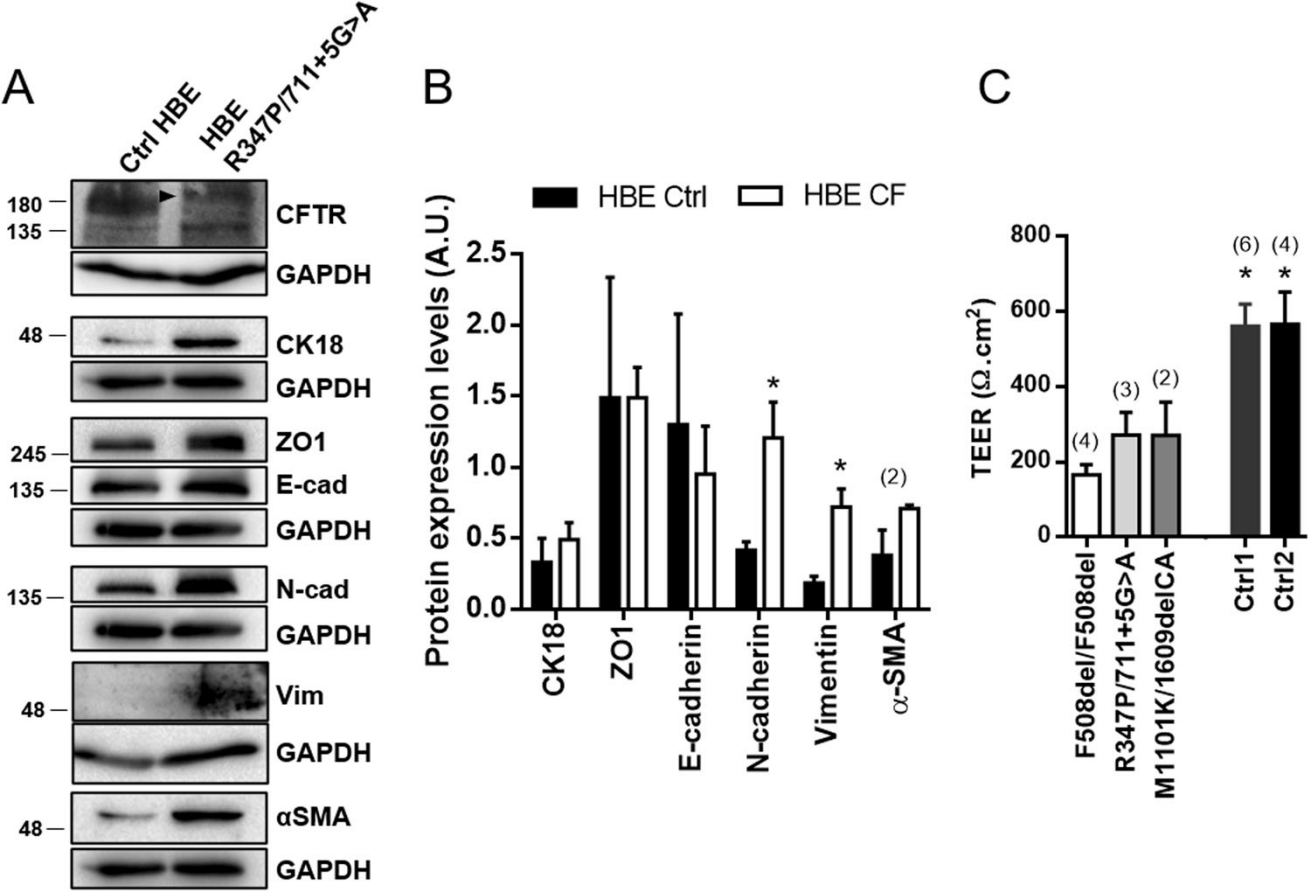
Primary CF HBE cells show increased expression levels of mesenchymal markers and impaired TEER. **A** Western blots showing epithelial (CFTR, CK18, ZO1, E-cadherin) and mesenchymal (N-cadherin, vimentin, αSMA) protein levels in fully differentiated Ctrl and CF (R347P/711 + 5 G > A) pHBE cells (21d of differentiation). In the R347P/711 + 5 G > A pHBE cells, a faint band C can still be seen for CFTR (arrowhead). GAPDH was used as a loading control. **B** Quantification by densitometry of the protein expression detected by WB in **A**. Data is normalized to loading control and showed as arbitrary units (A.U.), mean ± SEM. Asterisk indicates significant difference between Ctrl and CF pHBE cells (unpaired *t*-test, *p* < 0.05). The CF individuals’ genotypes were F508del/F508del, R347P/711 + 5 G > A and M1101K/1609delCA. (*n* = 3, unless indicated by (2)). **C** TEER measurements of CF and controls pHBE cells after 21 days in ALI. CF individuals’ genotypes were F508del/F508del, R347P/711 + 5 G > A and M1101K/1609delCA. Asterisk indicates significant difference between Ctrl and CF pHBE cells (unpaired *t*-test, *p* < 0.05). The number of filters used in the statistical analysis is indicated above each bar.

Next, we examined EMT in CF primary (p)HBE cells, which are the gold standard for physiological relevance in CF. WB assessing levels of epithelial/mesenchymal markers (Fig. 2 and S2) revealed no significant differences between CF and control pHBE cells regarding the epithelial proteins CK18, ZO-1 or E-cadherin (Fig. 2A, B). In contrast, mesenchymal markers N-cadherin and vimentin were significantly increased in CF vs control cells (Fig. 2A, B). A tendency towards increased α-SMA levels in CF cells was also detected (Fig. 2A, B).

Importantly, WB was performed on CF cells with different genotypes: F508del/F508del, R347P/711 + 5 G > A and M1101K/1609delCA (individual data in Fig. S2), confirming that the defects observed are characteristic of CF and not genotype-specific. For example, despite that R347P/711 + 5 G > A cells (Fig. 2A) express some mature, fully-glycosylated (albeit faint) band C, consistent with partially functional CFTR^35,36^, they display the same mesenchymal phenotype as other CF pHBE cells (Fig. 2 and S2). Notwithstanding, some variation occurred in the levels of epithelial proteins among CF cells with distinct CFTR genotypes (Fig. S2), suggesting that the latter may have an effect on these markers.

Similarly to CF tissue, pHBE CF cells show increased levels of mesenchymal markers, although not accompanied by decreased levels of epithelial markers. Consistently, TEER values (an indicator of integrity of HBE cell cultures^43^) were significantly lower in CF vs non-CF pHBE cells (Fig. 2C), indicative of a leaky epithelium and suggesting impaired organization of cell-cell junctions in CF.

### Polarized F508del-CFTR CFBE cells are more mesenchymal than wt-CFTR CFBE cells

To investigate how mutant CFTR affects junction organization we resourced to the polarized CFBE cell line overexpressing wt- or F508del-CFTR, which have the advantage of being isogenic, meaning that differences found between them can be directly linked to defective CFTR.

**Fig. 3:**
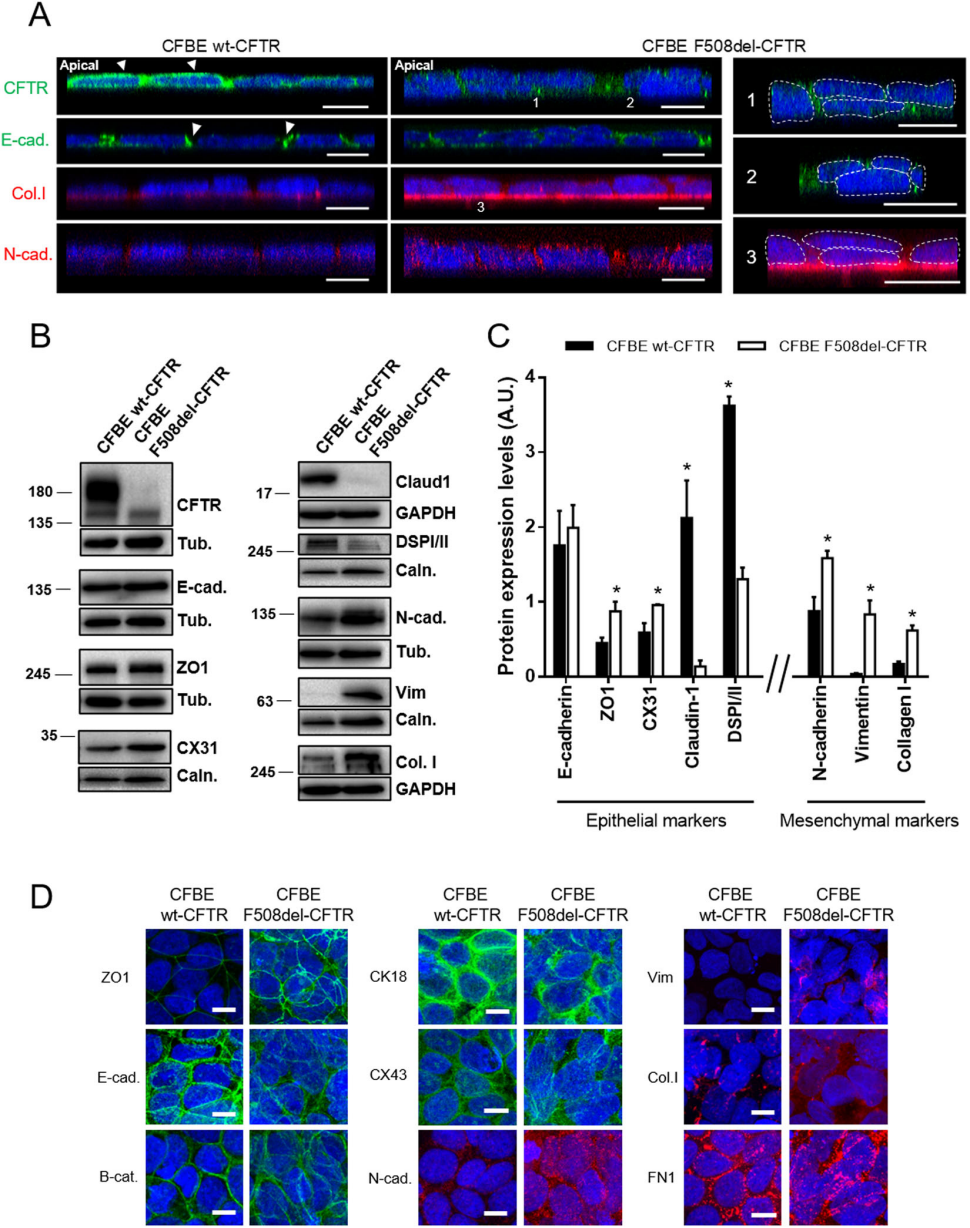
Polarized F508del-CFTR CFBE cells show mislocalized/disorganized epithelial markers and increased mesenchymal markers. **A** Z-stack of a representative group of cells showing the different localisation of CFTR, E-cadherin, collagen I and N-cadherin in polarized wt- and F508del-CFTR cells (xz/yz axis). A Leica TCS SP8 confocal microscope was used for image acquisition. Scale bar represents 10 μm. Epithelial markers display the correct localisation in wt-CFTR cells (arrowheads) but not F508del-CFTR cells. F508del-CFTR cells also display abolishment of a single cell monolayer with cells accumulating on top of each other (right panel, 1, 2, 3). The apical side of the epithelia is identified, and it is the same in all pictures. (*n* = 3) **B** Representative Western blots showing several epithelial (CFTR, E-cadherin, ZO1, CX31, claudin-1, DSPI/II) and mesenchymal (N-cadherin, vimentin, collagen I) protein levels in polarized wt- and F508del-CFTR CFBE cells. Calnexin, tubulin and GAPDH were used as loading controls. **C** Quantification by densitometry of the protein expression detected by WB in **B**. Data is normalized to loading control and showed as arbitrary units (A.U.), mean ± SEM. Asterisk indicates significant difference between wt- and F508del-CFTR cells (unpaired *t*-test, *p* < 0.05). (*n* = 3). **D** Assessment of the subcellular localisation of epithelial (ZO1, E-cadherin, β-catenin, CX43) and mesenchymal (N-cadherin, vimentin, collagen I and fibronectin) markers in polarized CFBE cells by immunofluorescence. A Leica TCS SP8 confocal microscope was used for image acquisition. Representative sections are shown as MIPs. Nuclei are depicted in blue and immunostained proteins in green (epithelial markers) or red (mesenchymal markers). Scale bar represents 10 μm. (*n* = 3).

Confocal microscopy (Fig. 3A), besides showing the expected different localisation of wt-CFTR (apical PM) and F508del-CFTR (intracellular)^44^, also revealed differences in the architecture of the epithelial cell cultures: while wt-CFTR cells formed a single cell monolayer, F508del-CFTR cells were multilayered, similarly to the CF lung pattern (Fig. 3A), indicative that the epithelial disorganization also occurs in airway epithelial cells expressing mutant CFTR. Consistently, E-cadherin was less confined to AJ in F508del-CFTR cells vs wt-CFTR cells and F508del-CFTR cells deposited more collagen I to the ECM and expressed increased levels of diffusely localized N-cadherin vs wt-CFTR cells (Fig. 3A).

To gain insight into how F508del-CFTR contributes to epithelial disorganization, we determined protein levels of EMT markers by WB in CFBE cells. As expected, CFTR was detected as mature and immature forms (bands C and B, respectively) in wt-CFTR expressing cells while only immature form was observed in F508del-CFTR cells (Fig. 3B). E-cadherin levels did not differ between these cells (Fig. 3B, C), nor did those of epithelial markers occludin, β-catenin, CX26, CX43 or CK18 (Fig. S3). However, ZO-1 and CX31 were increased while claudin-1 and DSPI/II were significantly decreased in F508del- vs wt-CFTR cells. The mesenchymal markers N-cadherin, vimentin and collagen I were all significantly increased in F508del-CFTR cells (Fig. 3B, C), while no significant changes were observed for αSMA and fibronectin (Fig. S3).

Further IF studies revealed localisation differences for several EMT markers in F508del-CFTR cells, similarly to lung tissue. ZO-1 staining was more intense in F508del-CFTR cells, while highlighting the disorganization of the epithelial cells and their multi-layer growth (Fig. 3D). E-cadherin and β-catenin stainings were more diffuse and not as concentrated at AJ in F508del-CFTR cells as in wt-CFTR cells. CK18 and CX43 were also more disorganized in F508del-CFTR cells, while retaining their normal localisation at the intermediate filaments and GJ, respectively, in wt-CFTR cells (Fig. 3D). N-cadherin, vimentin and collagen I were all increased in F508del- vs wt-CFTR cells, confirming the WB data (Fig. 3B–D). FN1 showed a more diffuse pattern in F508del-CFTR cells (Fig. 3D), consistently with the lung tissue. In line with this, TEER measurements demonstrated lower resistance values in F508del-CFTR cells vs. wt-CFTR cells (Fig. S4A).

Altogether, these data agree with lung tissue and pHBE cells findings and suggest that: (i) CFTR is needed for proper airway epithelial differentiation; (ii) mutant CFTR promotes partial EMT; and (iii) absence of functional CFTR alone (even without inflammation) is enough to drive EMT.

### CF cells show increased proliferation and decreased wound repair rates

**Fig. 4:**
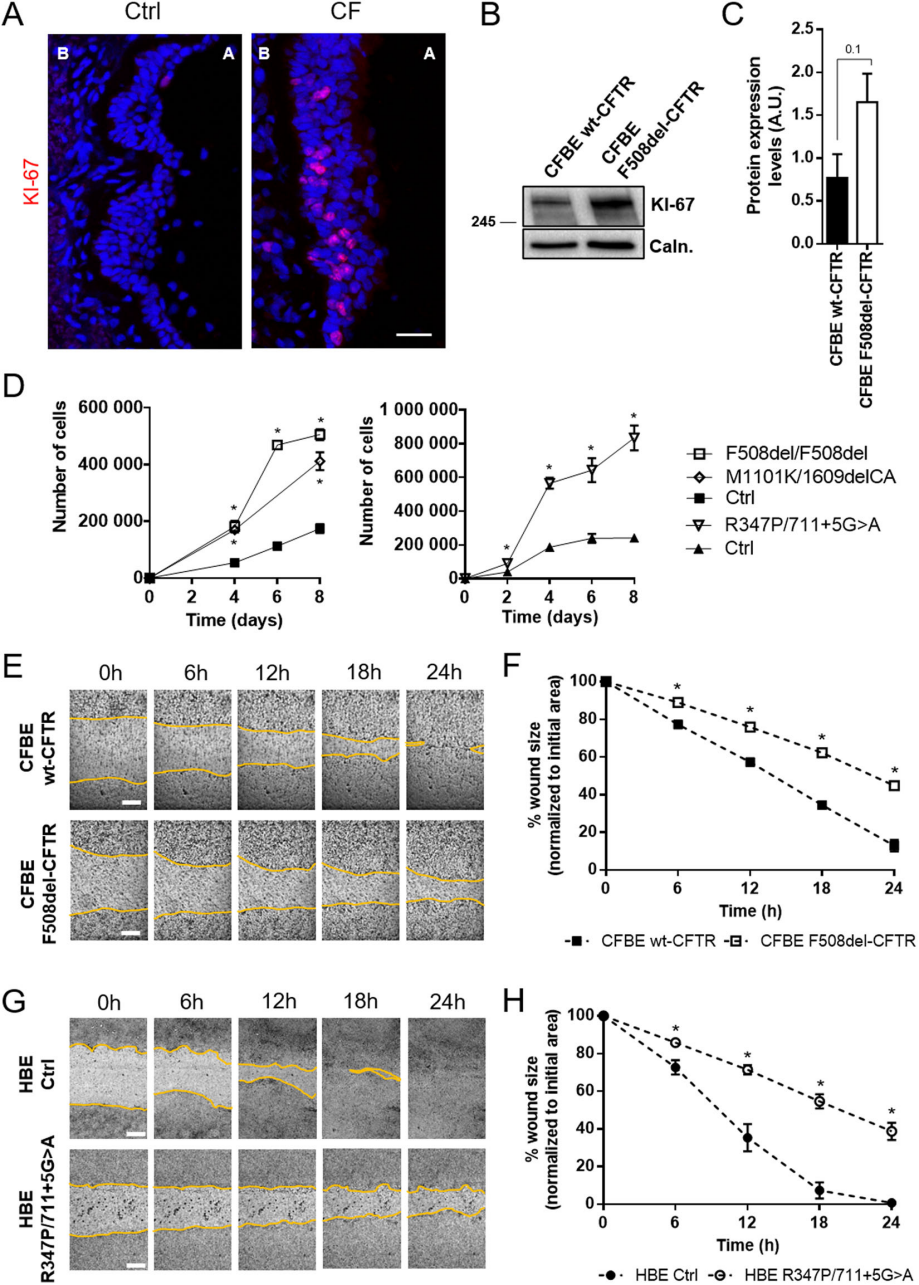
Absence of functional CFTR results in increased proliferation and decreased regeneration rates. **A** Representative images of native human bronchial tissue (Ctrl and CF) immunostained for Ki-67. (in red). Scale bar represents 25 μm. A great number of Ki-67-positive cells is seen in CF tissue when compared to control. Apical and basal (A and B respectively) sides of the epithelia are identified. Images are displayed as maximum image projections (MIPs). The CF lung in this figure had a R347P/711 + 5 G > A genotype, but similar findings were obtained in lungs with a F508del/F508del genotype. (*n* = 3). **B** Representative Western blot showing Ki-67 protein levels in polarized wt- and F508del-CFTR CFBE cells. Calnexin was used as loading control. **C** Quantification by densitometry of the protein expression detected by WB in **B**. Data is normalized to loading control and showed as arbitrary units (A.U.), mean ± SEM (unpaired *t*-test, *p*-value = 0.1). (*n* = 3) **D** Growth curve on non-polarized pHBE cells. (genotypes F508del/F508del, R347P/711 + 5 G > A and M1101K/1609delCA). Cells were harvested and counted every 2–4 days to assess the growth rate. The two different graphs represent experiments performed independently with different controls. Data is showed as cell number over 8 days, mean ± SEM. Asterisk indicates significant difference between Ctrl and CF pHBE cells (unpaired *t*-test, *p* < 0.05). (*n* = 3). **E**, **G** Representative images of scratch wounds over 24 h on **E** polarized CFBE cells and **G** fully differentiated pHBE cells. Live-cell imaging was performed on a Leica DMI6000B microscope with 37 °C and 5% CO_2_. Wounds segmentation is showed in yellow for clearer viewing. Scale bar represents 300 μm. **F**, **H** Wound closure analysis of **E**, **G** respectively. Data is represented as a percentage of the initial area of the wound, mean ± SEM. Asterisk indicates significant difference between F508del-CFTR and wt-CFTR CFBE cells or R347P/711 + 5 G > A and Ctrl pHBE cells (unpaired *t*-test, *p* < 0.05). (*n* = 5).

Next, experiments were performed to investigate the role of CFTR in cell proliferation and regeneration. Proliferation marker Ki-67 staining demonstrated a higher number of Ki-67-positive cells in basal cell layers of native CF airway epithelium vs control tissue (Fig. 4A). Polarized CFBE cells showed a trend (albeit not significant) towards higher Ki-67 expression in F508del-CFTR vs. wt-CFTR cells (Fig. 4B, C). The most striking results, however, were observed for pHBE cells (three different CFTR genotypes) exhibiting 3-fold higher cell proliferation rates vs control cells (Fig. 4D).

We investigated CFTR role in epithelial regeneration by wound-healing assays which showed that wt-CFTR-expressing cells closed the wounds 1.5–2 times faster than CF cells, for both polarized CFBE and fully differentiated pHBE cells, respectively (Fig. 4E–H). Thus, despite that CF cells proliferated at higher rates, they were significantly less capable of closing the wound and regenerating an intact epithelial cell layer. Impaired epithelial regeneration after wound closure in CF was also confirmed by the low TEER values measured in CFBE cells after wound healing live-cell imaging (Fig. S4B). In accordance with the above data, wt-CFTR cells recovered TEER values much faster after wounding than CF cells, being their resistance almost back to normal 48 h after wounding, while F508del-CFTR cells still had very low resistance 48 h post-wounding, only fully recovering 96 h after wounding. These data further emphasize the central role of CFTR in epithelial differentiation and regeneration.

### Functional CFTR restores a more epithelial phenotype and confers resistance to TGF-β1-induced EMT

**Fig. 5:**
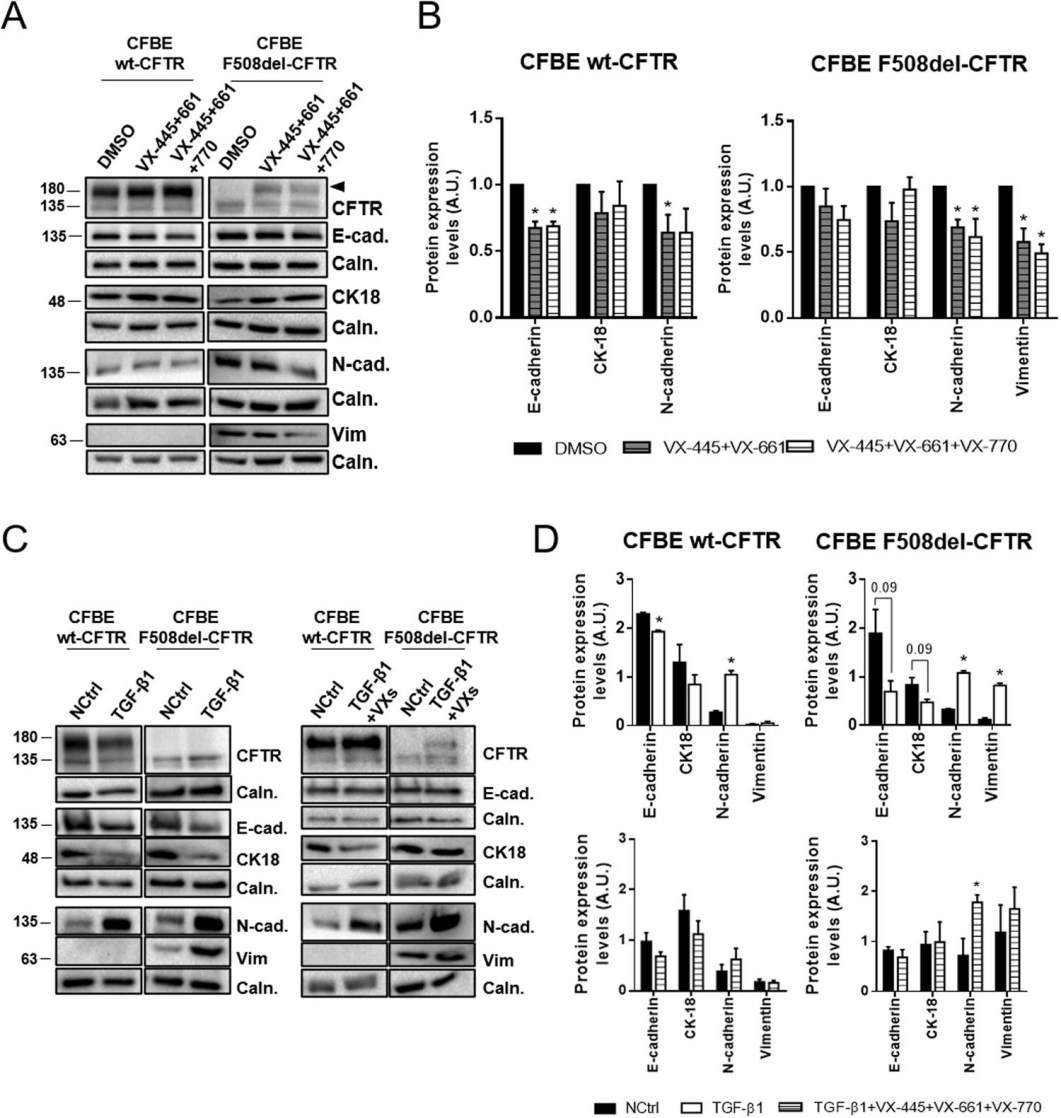
Functional CFTR can restore a more epithelial phenotype and confers resistance to TGF-β1-induced EMT. **A** Representative Western blots showing the effects of VX-445, VX-661 and/or VX-770 on CFTR, E-cadherin, N-cadherin, CK18 and vimentin in polarized CFBE cells. Calnexin was used as a loading control. Treatment with correctors VX-445 and VX-661 rescues F508del-CFTR increasing the amount of band C (arrowhead). **B** Quantification by densitometry of the protein expression detected by WB in **A**. Data is normalized to loading control and to negative control (DMSO) and showed as arbitrary units (A.U.), mean ± SEM. Asterisk indicates significant difference between negative control and treatment (unpaired *t*-test, *p* < 0.05). (*n* = 4). **C** Representative Western blots showing the effects of TGF-β1 and TGF-β1 with VX-445, VX-661 and VX-770 on CFTR, E-cadherin, N-cadherin, CK18 and vimentin in polarized CFBE cells. Calnexin was used as a loading control. **D** Quantification by densitometry of the protein expression detected by WB in **C**. Data are normalized to loading control and showed as arbitrary units (A.U.), mean ± SEM. Asterisk indicates significant difference between negative control and treatment (unpaired *t*-test, *p* < 0.05). (*n* = 3–4).

Since data above pointed to the absence of functional CFTR driving EMT, next we evaluated whether rescuing F508del-CFTR could revert the EMT phenotype. To this end, CFBE cells were treated with a highly effective CFTR modulator (HECM) drug, the most efficient CFTR corrector to date^45^, i.e., triple combo of two correctors (VX-445, VX-661) and a potentiator (VX-770). F508del-CFTR cells showed a clear correlation between the efficacy of mutant CFTR correction (and potentiation) and reduction in mesenchymal marker levels. VX-661 treatment, which only partially rescues F508del-CFTR (Fig. S5A), did not cause a significant change in the levels of EMT markers (Fig. S5B). The VX-661/VX-770 combination is already sufficient to significantly decrease vimentin levels by ~50% (Fig. S5B). However, when VX-661/VX-445 is added to F508del-CFTR cells, the correction is much more efficacious (Fig. 5A) and sufficient to significantly reduce the levels of both N-cadherin and vimentin by 25–50% (Fig. 5B), an effect that is even greater upon combination with potentiator VX-770. These data show that the amount of functional CFTR is closely correlated with the epithelial/mesenchymal status of these cells. As expected, wt-CFTR cells showed no major differences in levels of epithelial or mesenchymal markers when treated with these compounds (Fig. 5A, B and S5), although the AJ proteins (E-cadherin and N-cadherin) appear to be slightly sensitive to the addition of VX-445.

These results suggest that CFTR plays an important role in protecting against EMT induction. To further test this hypothesis, we exposed polarized CFBE cells to TGF-β1, which is overexpressed in CF tissues and has been shown to promote EMT in cultured airway epithelial cells^46^. Both wt- and F508del-CFTR expressing CFBE cells showed a similar decrease in CK18 and increase in N-cadherin expression in response to TGF-β1 (Fig. 5C, D). In contrast, E-cadherin levels were decreased by 63% in F508del-CFTR cells but only by ~15% in wt-CFTR cells (Fig. 5D). The levels of vimentin were increased by 10-fold in F508del-CFTR cells upon TGF-β1 treatment, while remaining undetectable in wt-CFTR cells. These results show that wt-CFTR expressing cells are more resistant to EMT induction than CF cells. However, TGF-β1 treated F508del-CFTR cells together with VX-445/VX-661/VX-770, shows no decreased levels of epithelial markers E-cadherin and CK-18 nor increased mesenchymal marker vimentin (Fig. 5C, D). F508del-CFTR rescue thus partially blocks the TGF-β1-mediated EMT induction. Moreover, treatment of wt-CFTR expressing cells with this HEMC completely blocked TGF-β1-induced EMT (Fig. 5C, D).

Altogether, these data provide evidence for a direct correlation between EMT and dysfunctional CFTR.

### Mutant CFTR drives TWIST1 mediated EMT

**Fig. 6:**
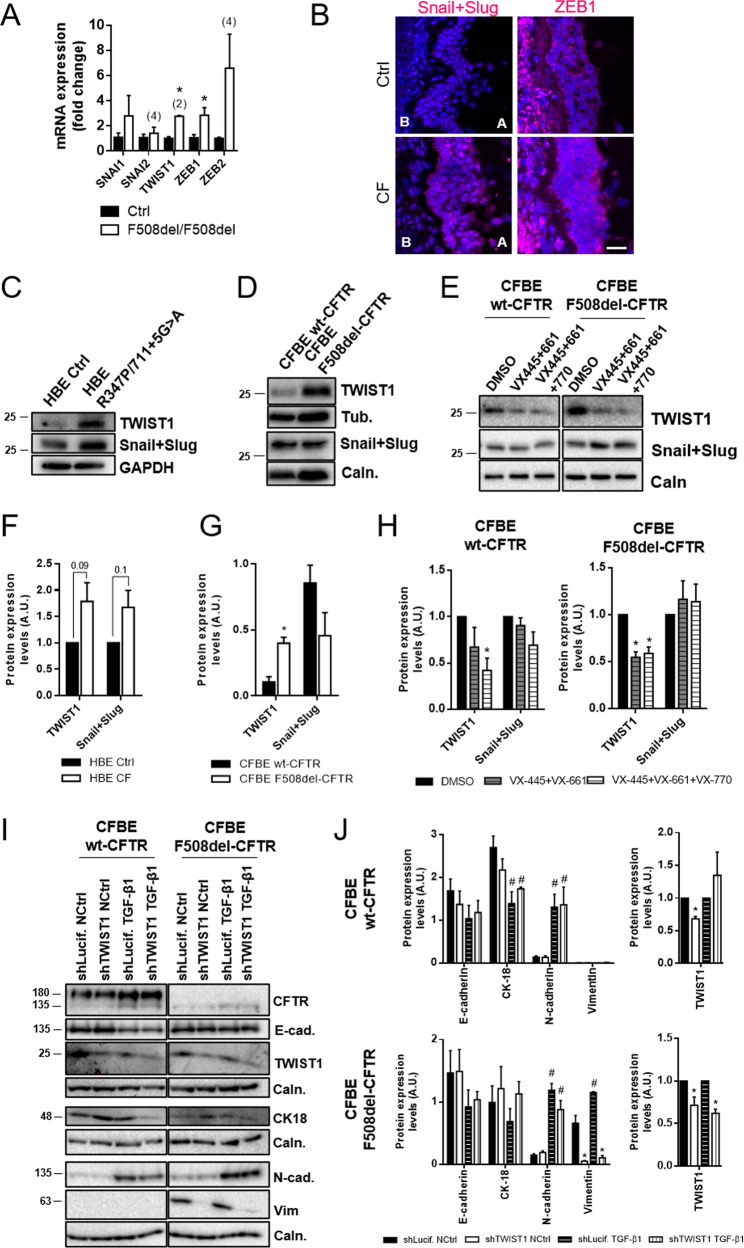
EMT-inducing transcription factors (EMT-TFs) are increased in the absence of functional CFTR, with TWIST1 mediating mutant CFTR driven EMT. **A** Transcript analysis of EMT-TFs levels by qRT-PCR on different control and F508del homozygous lung samples. Fold-change values were calculated by applying the ΔΔCT method and are represented by mean ± SEM relative to the control samples (*n* = 3, unless indicated by (2) or (4)). Asterisk indicates significant difference between F508del/F508del and Ctrl samples (unpaired t-test, *p* < 0.05). **B** Representative images of native human bronchial tissue (Ctrl and CF) immunostained for Snail + Slug and ZEB1. Nuclei are depicted in blue and the TFs in magenta. Scale bar represents 25 μm. Increased Snail + Slug and ZEB1 levels are present in CF tissues when compared to controls. Apical and basal (A and B respectively) sides of the epithelia are identified and are the same in all pictures. Images are displayed as maximum image projections (MIPs). The tissues stained display secondary/tertiary bronchi and were as similar as possible for comparison. The CF lung in this figure had a R347P/711 + 5 G > A genotype, but similar findings were obtained in lungs with a F508del/F508del genotype. Several controls were also assessed with similar results between themselves. (*n* = 2–3 samples). **C**, **D** Representative western blots showing Snail + Slug and TWIST levels on **C** fully differentiated pHBE cells and **D** polarized CFBE cells. Calnexin, tubulin or GAPDH were used as loading controls. **E** Representative Western blots showing the effects of VX-445, VX-661 and/or VX-770 on TWIST1 and Snail + Slug in polarized CFBE cells. Calnexin was used as a loading control. **F** Quantification by densitometry of the protein expression detected by WB in **C**. Data is normalized to loading control and to Ctrl cells and showed as arbitrary units (A.U.), mean ± SEM. The CF individuals’ genotypes were F508del/F508del, R347P/711 + 5 G > A and M1101K/1609delCA. (*n* = 3). **G** Quantification by densitometry of the protein expression detected by WB in **D**. Data is normalized to loading control and showed as arbitrary units (A.U.), mean ± SEM. Asterisk indicates significant difference between wt-CFTR and F508del-CFTR cells (unpaired *t*-test, p < 0.05). (*n* = 3). **H** Quantification by densitometry of the protein expression detected by WB in **E**. Data is normalized to loading control and to negative control (DMSO) and showed as arbitrary units (A.U.), mean ± SEM. Asterisk indicates significant difference between negative control and treatment (unpaired *t*-test, *p* < 0.05). (*n* = 4). **I** Representative Western blots showing the effects of TWIST1 knockdown on CFTR, E-cadherin, N-cadherin, CK18 and vimentin in polarized CFBE cells. Calnexin was used as a loading control. **J** Quantification by densitometry of the protein expression detected by WB in **I**. Data is normalized to loading control (for E-cad., CK18, N-cad. and Vim) or to loading control and negative control (TWIST1) and showed as arbitrary units (A.U.), mean ± SEM. Asterisk indicates significant difference between shLuciferase and shTWIST1, hash indicates significant difference between NCtrl and TGF-β1 (unpaired *t*-test, *p* < 0.05). (*n* = 3).

To elucidate which EMT pathways are involved in CF, expression of the main EMTa-TFs SNAIL1, SNAIL2, TWIST1, ZEB1 and ZEB2 was assessed. Transcript analysis in native lung tissue showed significant upregulation of TWIST1 and ZEB1 in CF vs non-CF tissue (Fig. 6A), while SNAIL1 and ZEB2 levels were also increased but not significantly. Consistently, positive staining for Snail + Slug and ZEB1 were found in CF but not control lung tissue (Fig. 6B). CF pHBE cells displayed increased levels of both TWIST1 and Snail + Slug (Fig. 6C, F), albeit not significant. Nevertheless, TWIST1 was significantly increased in F508del-CFTR CFBE cells (Fig. 6D, G). Since ZEB1 was not detectable by WB in CFBE cells (Fig. S6), TWIST1 upregulation might be the link to CFTR-mediated EMT. We thus treated F508del-CFTR cells with VX-445/VX-661/VX-770 and observed a significant reduction in TWIST1 levels but not in Snail + Slug levels (Fig. 6E, H). Interestingly, TWIST1 levels were only reduced significantly in wt-CFTR cells upon addition of potentiator VX-770 to VX-661/VX445 (Fig. 6H), reinforcing the importance of channel activity in EMT protection.

To further confirm the CFTR/ TWIST1 relationship, we knocked-down (KD) TWIST1 in CFBE cells using shRNA (~30–40% efficiency). TWIST1-KD was sufficient to inhibit vimentin expression both in the presence and absence of TGF-β1 in F508del-CFTR cells (Fig. 6I, J). Moreover, no significant changes were observed for TWIST1-KD in wt-CFTR cells (Fig. 6J), suggesting a CF-specific role for TWIST1.

## Discussion

The main goal of this study was to determine whether defective CFTR is linked to impaired epithelial cell differentiation and EMT activation in CF airways. Combining studies in human native lung tissue, primary and immortalized bronchial epithelial cells, we found evidence of abnormal epithelial–mesenchymal plasticity in CF tissues/cells expressing mutant CFTR, including mislocalisation of cell junction proteins, disruption of epithelial architecture, aberrant expression of mesenchymal markers and EMTa-TF upregulation.

While mesenchymal markers, including vimentin and N-cadherin, were consistently upregulated in CF tissues/cells, E-cadherin and most epithelial cell junction proteins were not repressed, except for claudin-1 (TJ component) and DSPI/II (desmosome component). This is consistent with partial EMT shown by multiple studies to occur under pathological conditions, e.g., cancer, fibrosis and other chronic lung diseases^25,28,47^. Indeed, increasing evidence indicates that pathological EMT is not a binary On/Off process but rather a spectrum of states, in which cells express both epithelial and mesenchymal phenotypes simultaneously^48^. Complete transition from an epithelial to mesenchymal phenotype may only apply to developmental EMT^25,49^. However, considering our finding that some EMTa-TFs (TWIST1, Snail + Slug and ZEB1) are upregulated in CF tissues/cells, it was somewhat surprising that mislocalisation of cell junction proteins and epithelial architecture disruption was only associated with downregulation of two junction proteins. In fact, some epithelial markers (ZO-1, CX31 and CK18) were upregulated in CF. The explanation for this can be two-fold. Firstly, our data clearly show that in CF tissue/cells epithelial proteins expression is accompanied by significant disorganization and showing abnormal subcellular localisation. It is likely that the described CF associated inappropriate formation of cell junctions^6,50,51^, which we also found here, can activate a positive feedback loop trying to compensate for the absence of functional junctions by upregulating epithelial markers. Secondly, and importantly, TWIST1 is a more potent mesenchymal inducer than epithelial repressor, whereas SNAIL1 and ZEB1 are strong epithelial repressors and weaker mesenchymal promoters^30^. We found TWIST1 to be the most consistently significantly increased EMTa-TF in CF tissues/cells, which is in line with the observed upregulation of mesenchymal markers without substantial repression of epithelial markers.

Data shown here clearly demonstrate a direct link between dysfunctional CFTR and TWIST1 upregulation, being correction of F508del-CFTR with HECM drug (VX-661/VX-445/VX-770) enough to significant revert TWIST1 expression to normal levels. Moreover, TWIST1 downregulation in F508del-CFTR expressing cells also partially reverts the increased sensitivity of these cells to TGF-β1-induced EMT while having no significant effect on wt-CFTR cells. This is additional proof that TWIST1 plays a key role in CF-specific EMT in the airways. This shows for the first time a link between dysfunctional CFTR and TWIST1-mediated EMT. Possible CFTR/TWIST1 bridging pathways include: (1) β-catenin, which interacts with both CFTR^52^ and TWIST1^53^ in EMT contexts; (2) transcription factor hypoxia-inducible factor 1α (HIF1α) implicated in CF pathophysiology^54^ and a known TWIST1 regulator^55^; and/or (3) NF-κB, an important player in CF^56^ also associated with TWIST1^57^.

Consistent to EMT being active in CF, our data also show that F508del-CFTR expressing cells are over-sensitive to TGF-β1-induced EMT vs non-CF cells. However, rescue of F508del-CFTR PM localisation and function by HECM drug partially blocked TGF-β1-mediated EMT induction. This emphasizes the important role that CFTR plays in preserving cell junction integrity and protecting against TGF-β1-induced EMT. Furthermore, rescue of F508del-CFTR in the absence of TGF-β1 led to a significant reduction of the expression of N-cadherin and vimentin, thus restoring a more epithelial phenotype, an effect that is even greater upon combination with potentiator VX-770. It seems thus probable that functional PM CFTR is required for the maintenance of the cell junction integrity and correct architecture of polarized epithelial cells. Others have also reported that functional PM CFTR is essential for epithelial tightness and normal organization and function of TJs^6^ and that it regulates TJ formation and maintenance^58,59^. Consistently, here decreased TEER values in CF cells were also observed, indicative of leaky epithelia and TJ defects. Those studies had not related EMT to absence of functional CFTR but actually TJ remodelling is an early, key EMT event^60^. TJs are not only points of cell-cell interactions but also hubs for intracellular pathways regulating various cellular processes including epithelial differentiation and integrity. Consistently, we recently found that the coxsackie- and adenovirus receptor (CXADR), a transmembrane component of TJs, protects against TGF-β1-induced EMT in epithelial cells by regulating AKT signalling^61^, which we also found to be dysregulated in CF^62^.

Although most studies focus on apical CFTR as essential for epithelial cell organization, we found that CFTR function might be equally important in maintaining epithelial tightness. Indeed, potentiator VX-770 had an important additive effect in EMT protection and TWIST1 levels were only reduced significantly in wt-CFTR expressing cells upon potentiation of CFTR activity. CFTR is needed to maintain the correct levels of intracellular Cl^−^ ([Cl^−^]_i_), described to play a role as second messenger affecting processes such as cell cycle, cell proliferation and differentiation, among others^63^. It is possible that abnormal [Cl^−^]_i_ is in the origin of the EMT observed here in CF.

**Fig. 7:**
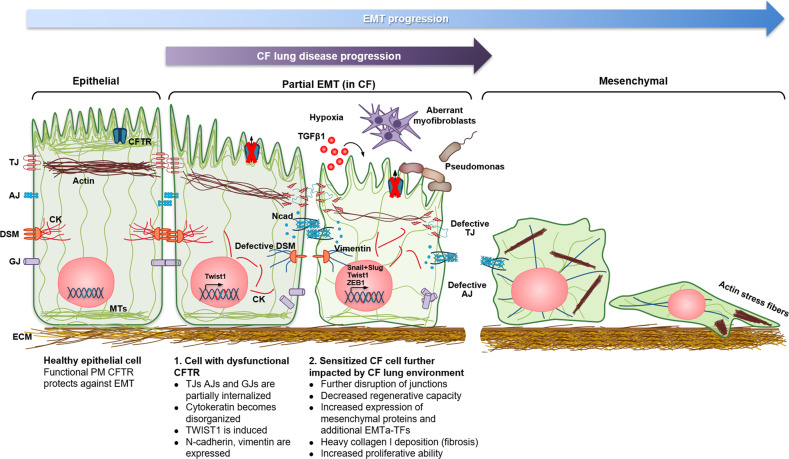
Epithelial defects in CF airways occur as a ‘two-hit’ event. In healthy airway epithelial cells functional PM CFTR has a role in protecting against EMT. However, when dysfunctional CFTR is present in these cells a shift occurs towards a less differentiated state, and the cells are more susceptible to EMT upon EMT stimuli (e.g. TGF-β1). A partial EMT occurs with cells losing their normal epithelial architecture while increasing levels of both mesenchymal markers and EMTa-TFs. Upon progression of CF lung disease, the lungs of CF individuals present the perfect mixture of conditions (e.g. constant inflammatory factors, such as TGF-β1, hypoxia, aberrant myofibroblast persistence and invasion by pathogens such as *Pseudomonas*) for further aberrant differentiation and enhanced EMT over time. (CK cytokeratin; DSM desmosomes; IF intermediate filaments; MTs microtubules; Ncad N-cadherin).

Altogether, our data suggest that absence of functional CFTR triggers TWIST1-mediated partial EMT. Moreover, since rescuing mutant CFTR with HECM drugs reverted EMT, CFTR likely plays a direct role in EMT protection. We thus propose that CFTR-mediated anion transport (possibly Cl^−^) is the driver of signalling which are essential for epithelial cell differentiation, TJ integrity and EMT protection (Fig. 7). However, in CF, proteins that would normally be confined to cell junctions in response to apical functional CFTR lose their correct localisation or reduce expression levels. In parallel to EMT, absence of functional PM CFTR can also activate signalling pathways normally active in undifferentiated cells, like cell proliferation. Hyperproliferation and impaired wound healing were here confirmed as part of CF pathology^3,7,8,12^ occurring in conjunction with EMT. The activation of different signalling pathways by F508del- and wt-CFTR is supported by the knowledge that their interactomes differ^64^ (also shown here for TWIST1), suggesting that dysfunctional CFTR translates into dysregulation of multiple signalling pathways. These data may appear contradictory, since usually hyperproliferative and migratory/invasive behaviours are seen as characteristic of EMT. However, epithelial-like and mesenchymal-like cells can migrate differently, the former migrating within a confluent monolayer where cell-to-cell contacts are maintained and the latter migrating as individual cells^65^. In the ‘partial’ CF EMT, cells still migrate mostly as monolayers as observed by live-cell imaging of wound closure. Thus, the CF TJ defects could account for the observed CF delay in wound closure, given the importance of cell-to-cell contacts in cell migration. In parallel, mutant CFTR can cause increased proliferation while being detrimental to tissue regeneration, as reported^3,8^. Moreover, migration is not exclusive of invasiveness and it has been shown to be required (via Notch activation) for normal wound healing^66^, probably the case here. In fact, some authors report that increased migration does not necessary occur in EMT^67^.

Based on our findings, we propose that epithelial defects in CF occur as a ‘two-hit’ event (Fig. 7). Firstly, absence of functional CFTR (regardless of genotype and without inflammation) causes a shift in epithelial cells towards a more proliferative, less differentiated state. This shift is accompanied (led?) by induction of partial EMT, with cells losing their normal epithelial architecture and upregulating both mesenchymal markers and TWIST1. Secondly, once the epithelial phenotype is ‘weakened’, its intrinsic resilience is also lost, leading to less resistance to EMT, which is potentiated by known hallmarks of CF lung disease: chronic inflammation, i.e. increased TGF-β1^29^, epithelial remodelling, pathogen invasion^3^, hypoxia and aberrant myofibroblast persistence^68^. These manifestations prime the CF lungs for further aberrant differentiation and enhanced EMT (e.g. increased mesenchymal proteins and other EMTa-TF like Snail + Slug and ZEB1) over time. Altogether, both ‘hits’ help to explain the occurrence of fibrosis and tissue degeneration and probably also cancer in CF.

With the increase of life expectancy in CF^69^, previously underlying aspects of the disease, such as fibrosis and high cancer prevalence, are likely to emerge, thus requiring novel therapeutic strategies. Targeting EMT might be a fruitful approach to preserve epithelial integrity and protect against developing cancer and fibrosis. On the other hand, this work also sheds some light on the role of CFTR as a protector of EMT and tumour suppressor, which can lead to novel avenues in the treatment/prevention of carcinogenesis.

## Supplementary information

Figure S1

Figure S2

Figure S3

Figure S4

Figure S5

Figure S6

Table S1

Table S2

Table S3

Supplementary information
