# Supplementary figures and images for: Mutant CFTR Drives TWIST1 mediated epithelial–mesenchymal transition
Source: Cell Death Dis. 2020 Oct 26;11(10):920. doi: 10.1038/s41419-020-03119-z (PMC7588414; doi:10.1038/s41419-020-03119-z)

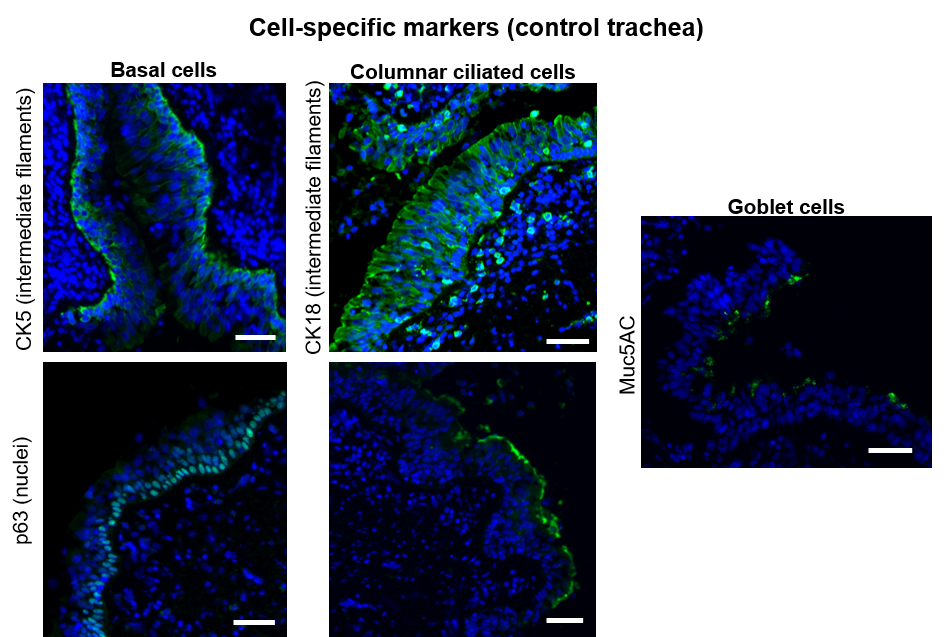

Supplement: Supplementary file 1 — Figure S1 [file 41419_2020_3119_MOESM1_ESM.tif]

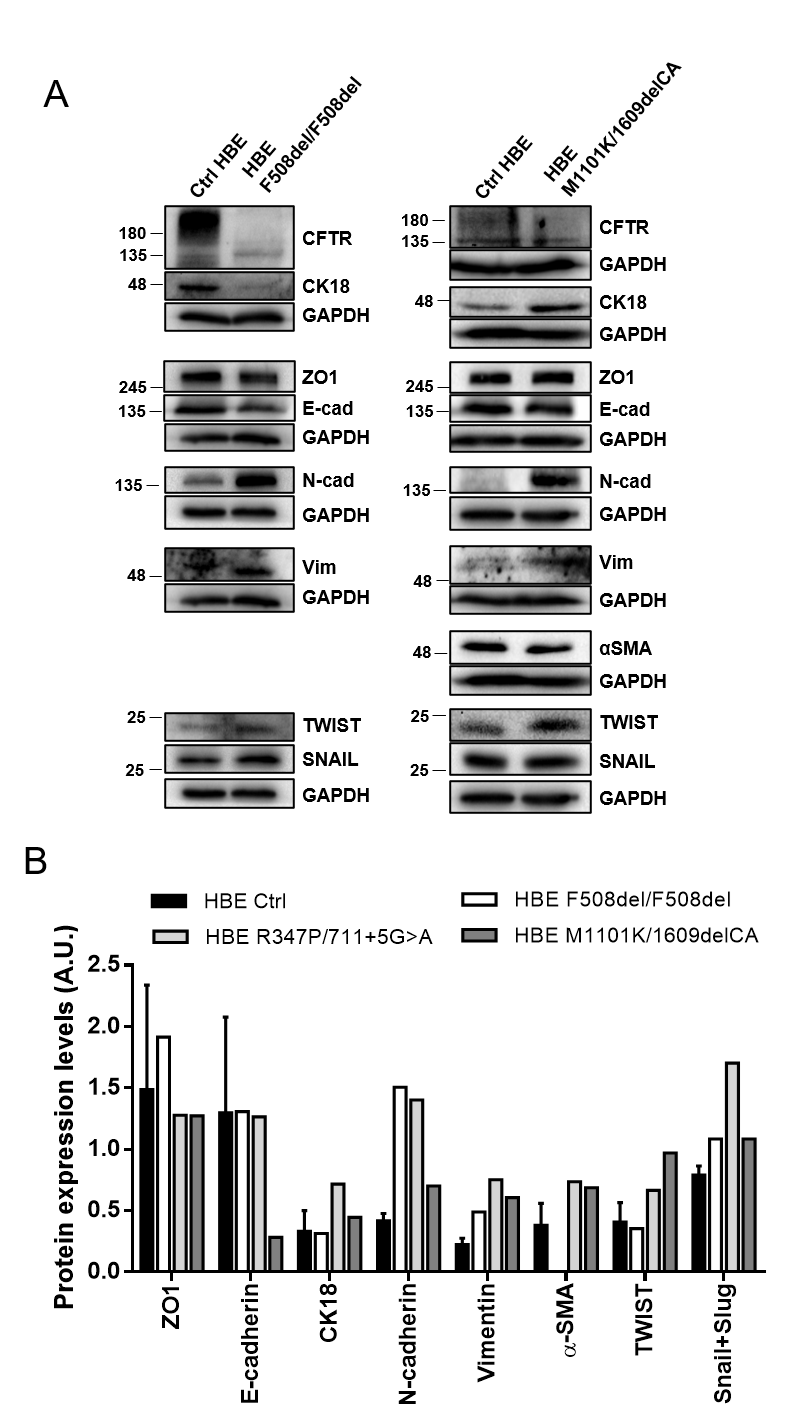

Supplement: Supplementary file 2 — Figure S2 [file 41419_2020_3119_MOESM2_ESM.tif]

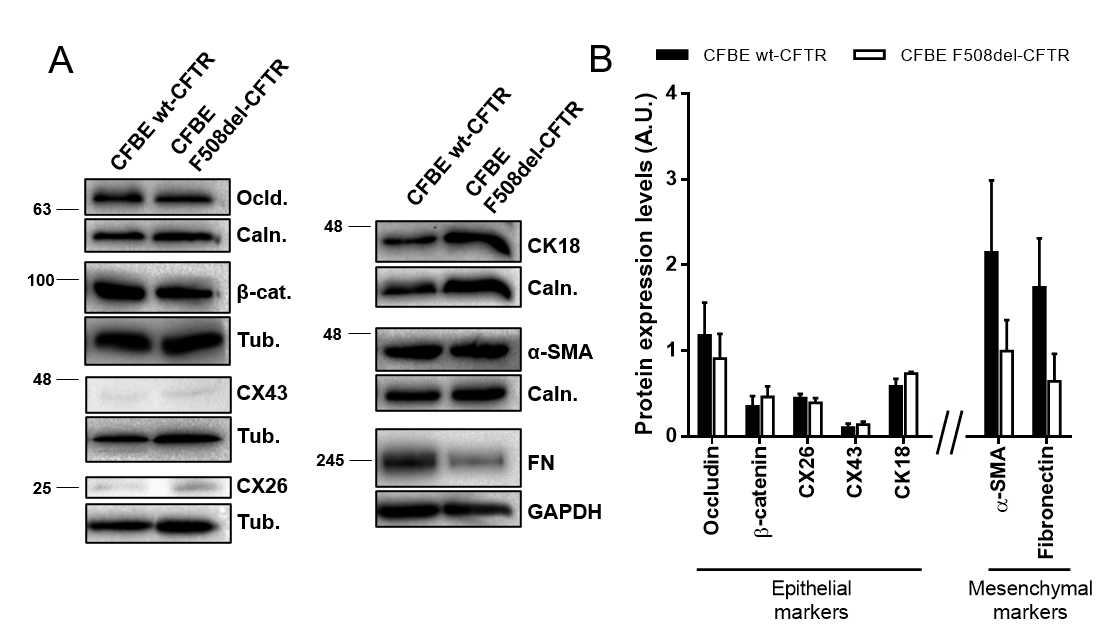

Supplement: Supplementary file 3 — Figure S3 [file 41419_2020_3119_MOESM3_ESM.tif]

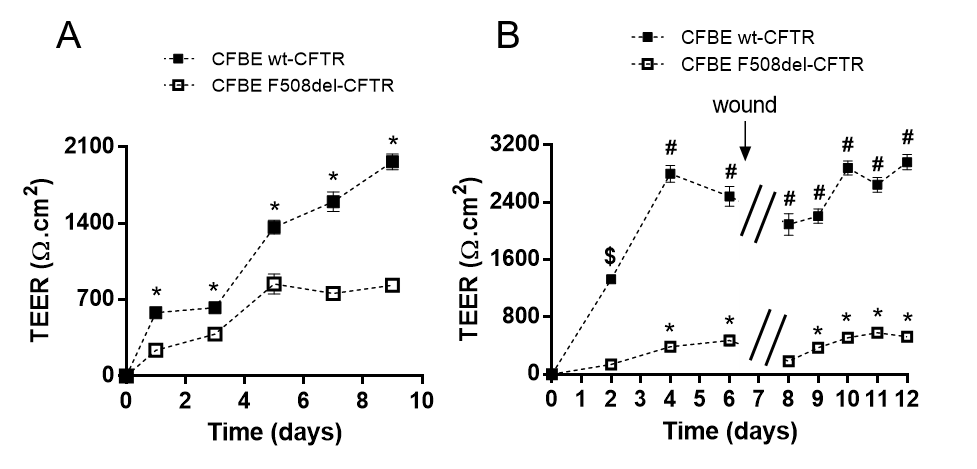

Supplement: Supplementary file 4 — Figure S4 [file 41419_2020_3119_MOESM4_ESM.tif]

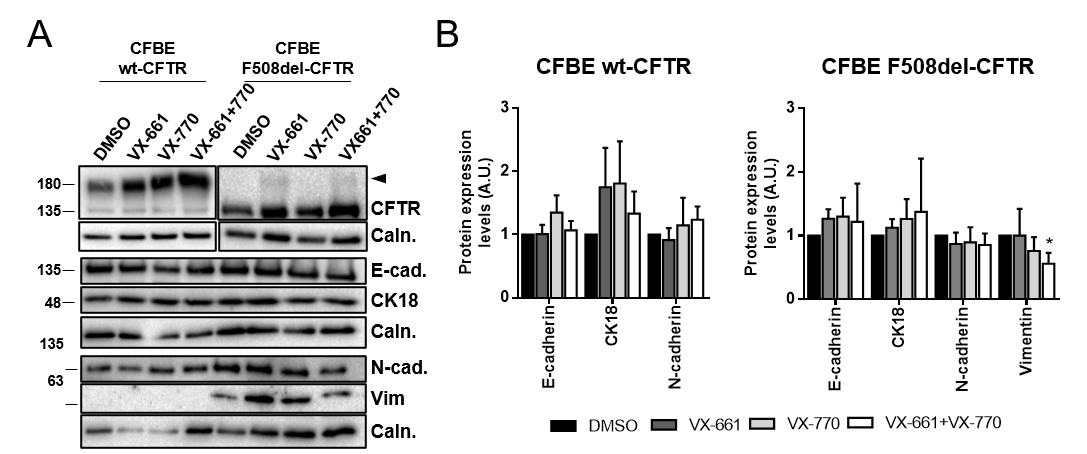

Supplement: Supplementary file 5 — Figure S5 [file 41419_2020_3119_MOESM5_ESM.tif]

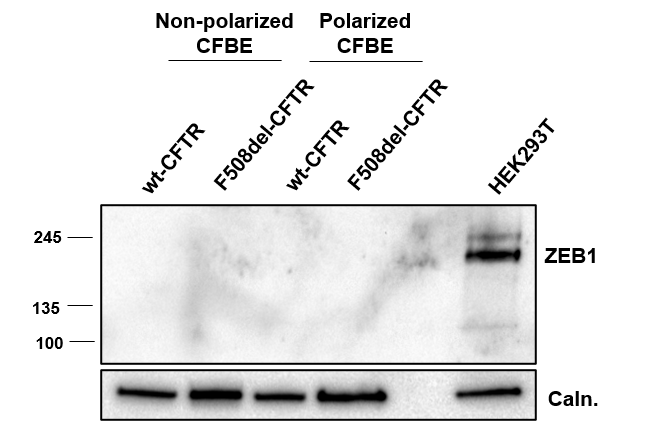

Supplement: Supplementary file 6 — Figure S6 [file 41419_2020_3119_MOESM6_ESM.tif]
