# Supplementary material for: Mutant CFTR Drives TWIST1 mediated epithelial–mesenchymal transition
Source: Cell Death Dis. 2020 Oct 26;11(10):920. doi: 10.1038/s41419-020-03119-z (PMC7588414; doi:10.1038/s41419-020-03119-z)
Supplement: Supplementary file 10 — Supplementary information [file 41419_2020_3119_MOESM10_ESM.docx]

**Supplementary Data**

**Table S1. Primary antibodies used.** Information like the experiment they were used in, the respective dilution, the host, the company and reference are clarified.

**Table S2. Secondary antibodies used.** Information like the experiment they were used in, the respective dilution, the host, the company and reference are clarified.

**Table S3. Description of the primers used in qRT-PCR.** Sequences for the primers were found at Harvard Primerbank.

**Figure S1. Cell specific markers detected by immunofluorescence in human airway tissue.** These results confirm the success of the current lung processing and cryopreserving protocol in the maintenance of the epitopes and overall architecture of the lung epithelia. Representative images of native human control tracheal tissue. Tissue was immunostained for cytokeratin 5 (intermediate filaments of basal cells), p63 (nuclei of basal cells), cytokeratin 18 (intermediate filaments of ciliated cells), tubulin IV (microtubules in cilia) and muc5ac (goblet cells). Nuclei were stained with Hoechst. Nuclei are depicted in blue and proteins in green. Scale bar represents 25μm. A Leica DMI6000B microscope was used for image acquisition. (CK5 Invitrogen PA1-37974 1:100; p63 Abcam ab124762 1:200; CK18 Santa Cruz sc-32329 1:100; Beta IV Tubulin Abcam ab11315 1:200; Muc5ac Invitrogen MA1-38223 1:200)

**Figure S2. Epithelial and mesenchymal protein levels on Ctrl and CF pHBE cells (all CF genotypes). (A)** Western blots showing epithelial (CFTR, CK18, ZO1, E-cadherin) and mesenchymal (N-cadherin, vimentin, αSMA) protein levels in fully differentiated Ctrl and CF (F508del/F508del and M1101K/1609delCA) pHBE cells (21d of differentiation). GAPDH was used as a loading control. **(B)** Quantification by densitometry of the protein expression detected by WB in (A). Data is normalized to loading control and showed as arbitrary units (A.U.), mean ± SEM for HBE Ctrl and individual value for the CF genotypes F508del/F508del, R347P/711+5G>A and M1101K/1609delCA.

**Figure S3. Epithelial and mesenchymal protein levels on polarized wt- and F508del-CFTR CFBE cells. (A)** Representative western blots showing several epithelial (occludin, β-catenin, CX26, CX43 and CK18) and mesenchymal (αSMA and fibronectin) protein levels. Calnexin, tubulin and GAPDH were used as loading controls. **(B)** Quantification by densitometry of the protein expression detected in (A). Data is normalized to loading control and showed as arbitrary units (A.U.), mean ± SEM. (n=3)

**Figure S4. TEER measurements (volt-ohmmeter) in polarizing wt- and F508del-CFTR CFBE cells. (A)** CFBE cells differentiating over time. Cells were maintained as submerged cultures. Cells were seeded on day 0. FBS was switched for 10% to 2% on day 1. Data is represented as Ω.cm^2^ over 9d. * indicates significant difference between wt-CFTR and F508del-CFTR cells (unpaired t-test, p<0.05). (n=4) **(B)** TEER measurements of the polarized CFBEs before and after wound. Wound was performed on day 6 after seeding, as indicated. Data is represented as Ω.cm^2^ over 12d. * indicates significant when compared to day 2 of same cell line, $ indicates significant when compared to F508del-CFTR, # indicates significant when compared to day 2 and F508del-CFTR (p<0.01). (n=5-7)

**Figure S5. CFTR correction can partially restore a more epithelial phenotype (A)** Representative western blots showing the effects of VX-661 and/or VX-770 on CFTR, E-cadherin, N-cadherin, CK18 and vimentin in polarized CFBE cells. Calnexin was used as a loading control. Treatment with corrector VX-661 rescues F508del-CFTR increasing the amount of band C (arrowhead). **(B)** Quantification by densitometry of the protein expression detected by WB in (A). Data is normalized to loading control and to negative control (DMSO) and showed as arbitrary units (A.U.), mean ± SEM. * indicates significant difference between negative control and treatment (unpaired t-test, p<0.05). Other p-values are indicated on the graphs. (n=4-5)

**Figure S6. CFBE cells do not express ZEB1 at detectable levels.** Representative Western blot performed on non-polarized and polarized wt- and F508del-CFTR CFBE cells, as well as HEK293T cells. Calnexin was used as loading control. (n=3)
